# ﻿Taxonomic review of the subgenus Tatsipolia Benedek, Behounek, Floriani & Saldaitis of the genus *Dasypolia* Guenée with descriptions of two new species from southern Xizang, China (Insecta, Lepidoptera, Noctuidae)

**DOI:** 10.3897/zookeys.1115.84527

**Published:** 2022-08-03

**Authors:** Enyong Chen, Zhaohui Pan, Anton V. Volynkin, Aidas Saldaitis, Balázs Benedek

**Affiliations:** 1 Key Laboratory of Forest Ecology in Tibet Plateau (Institute of Plateau Ecology, Tibet Agricultural and Animal Husbandry University), Ministry of Education, Linzhi 860000, China Tibet Agricultural and Animal Husbandry University Linzhi China; 2 Altai State University, Lenina Avenue, 61, RF-656049, Barnaul, Russia Altai State University Barnaul Russia; 3 Nature Research Centre, Akademijos str., 2, LT-08412, Vilnius-21, Lithuania Nature Research Centre Vilnius Lithuania; 4 H-2045 Törökbálint, Árpád u. 53, Hungary Unaffiliated Törökbálint Hungary

**Keywords:** Antitypina, new record, Noctuinae, Owlet moth, systematics, taxonomy, Xylenini

## Abstract

The subgenus Tatsipolia Benedek, Behounek, Floriani & Saldaitis, 2011 of the genus *Dasypolia* Guenée, 1852 is reviewed. Two new species, D. (T.) sejilaensis**sp. nov.** and D. (T.) cerritula**sp. nov.** are described from the Linzhi (Nyingchi) Prefecture in southern Xizang, China. The adults and the male and female genitalia of all species in the subgenus are illustrated. Additionally, Dasypolia (Auropolia) carlotta Floriani, Benedek, Behounek & Saldaitis, 2011 is reported from Xizang for the first time.

## ﻿Introduction

*Dasypolia* Guenée, 1852 is a large noctuid genus distributed in the Palearctic region and reaching its greatest diversity in high mountain areas of Asia. The genus belongs to the subtribe Antitypina of the tribe Xylenini of the subfamily Noctuinae (Lafontaine and Schmidt 2010; Zahiri et al. 2011; Keegan et al. 2021). Many species of *Dasypolia* were described during the last two decades (Nupponen and Fibiger 2006; Ronkay et al. 2010; Benedek et al. 2011, 2014; Volynkin 2012; Benedek and Saldaitis 2014; Ronkay et al. 2014). However, it is likely that additional undescribed species will be found.

The subgenus Tatsipolia Benedek, Behounek, Floriani & Saldaitis, 2011 was erected to solely include *D.ruficilia* Benedek, Behounek, Floriani & Saldaitis, 2011 (Benedek et al. 2011). Subsequently, another species of the genus, *D.vignai* L. Ronkay & Zilli, 1993 was assigned to *Tatsipolia* after the discovery of the male of the species (Benedek and Saldaitis 2014). During entomological studies in the southern Xizang Province of China short series of two unknown small *Dasypolia* specimens was collected by the senior and the second authors of the present paper. After comparing the male genitalia structures of these specimens with other species in the genus, they proved to belong to the subgenus Tatsipolia and express significant distinctive characters. The species are described herein as new to science.

## ﻿Materials and methods

Abbreviations for private and institutional collections used herein: **AFM** = collection of Alessandro Floriani (Milan, Italy); **HNHM** = Hungarian Natural History Museum (Budapest, Hungary); **TAAHU** = Tibet Agricultural and Animal Husbandry University (Linzhi, China); **ZSM** = Bavarian State Collection of Zoology (Zoologische Staatssammlung München, Munich, Germany). Other abbreviations used: **HT** = holotype; **PT** = paratype. In the type labels citations, different labels are separated by a slash (“/”) whereas the different lines of the same label are separated by an upright slash (“|”).

The male and female genitalia terminology follows Kononenko (2010).

## ﻿Results

**Noctuidae Latreille, 1809**

**Noctuinae Latreille, 1809**

**Xylenini Guenée, 1837**

**Antitypina Forbes & Franclemont, 1954**

### Genus *Dasypolia* Guenée, 1852

#### ﻿ Subgenus Tatsipolia

Taxon classificationAnimaliaLepidopteraNoctuidae

Benedek, Behounek, Floriani & Saldaitis, 2011

98613EE9-ED80-543A-AB80-B779A2238B2C

Dasypolia (Tatsipolia) Benedek et al. 2011: 108. Type species: Dasypolia (Tatsipolia) ruficilia Benedek, Behounek, Floriani & Saldaitis, 2011, by original designation.

##### Diagnosis.

Members of the subgenus are small moths (forewing length is 11-13 mm) externally similar to taxa of the subgenus Cteipolia. However, despite the external similarity, the subgenus is characterised by the following diagnostic features in the male genitalia: (1) The uncus is short but wide, triangular, dorso-ventrally flattened; (2) The harpe is reduced, tubercle- or spine-like; (3) The digitus is robust, thorn-like; (4) The juxta bears a medial process posteriorly; and (5) The phallus is relatively short but broad, with vesica bearing one or two clusters of spine-like cornuti. In the female genitalia, the broad ostium bursae and the sideways curved ductus and corpus bursae are characteristic for the subgenus.

##### Distribution.

Species of the subgenus are known only from south-western China (Sichuan and southern Xizang).

##### Species content of Dasypolia (Tatsipolia).

D. (T.) sejilaensis sp. nov.

D. (T.) cerritula sp. nov.

D. (T.) vignai L. Ronkay & Zilli, 1993.

D. (T.) ruficilia Benedek, Behounek, Floriani & Saldaitis, 2011.

#### ﻿ Dasypolia (Tatsipolia) sejilaensis sp. nov.

Taxon classificationAnimaliaLepidopteraNoctuidae

23868D91-BF18-5B20-A43F-DA414B94481C

https://zoobank.org/9AD848DE-FFAF-4D00-B6E2-CB8F8A2DACBE

**Figures 1–8.**
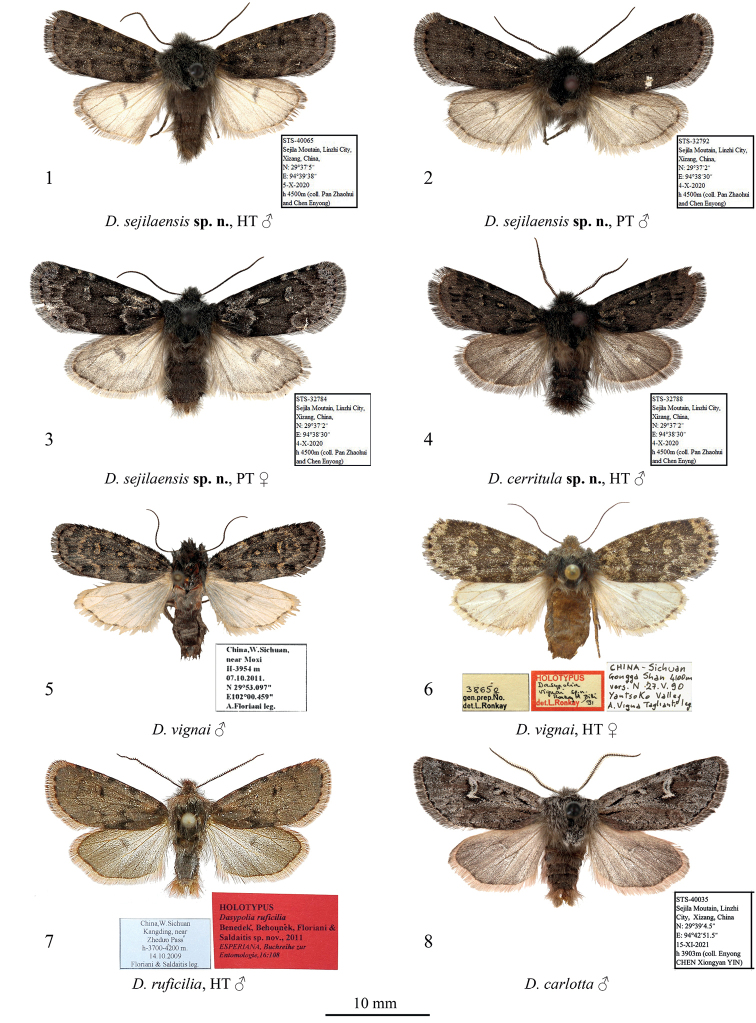
*Dasypolia* (*Tatsipolia* and *Auropolia*) spp., adults. Depositories of the specimens: **1–4** and **8** in TAAHU**5** in AFM**6** in HNHM (photo by B. Tóth) **7** in ZSM.

**Figures 9–11.**
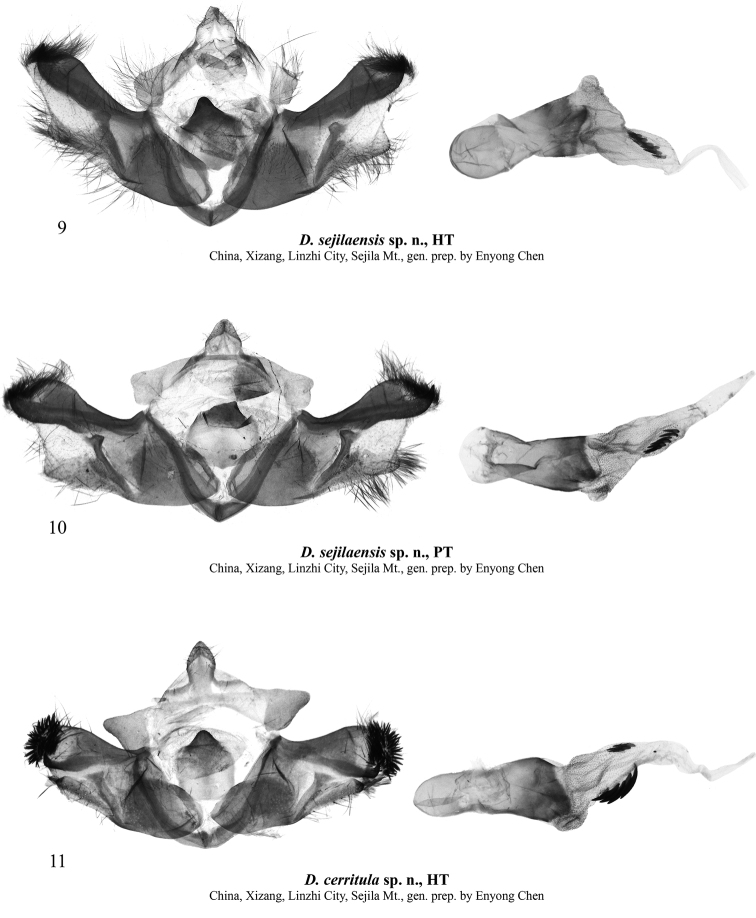
Dasypolia (Tatsipolia) spp., male genitalia. The specimens dissected are deposited in TAAHU.

Figs 1–3 , 9 , 10 , 15

##### Type material.

***Holotype*** (Figs 1, 9): male, “STS-40065 | Sejila Mountain, Linzhi City, | Xizang, China, | N:29°37'5" | E:94°39'38" | 5-X-2020 | h [Altitude] 4500 m (coll. Pan Zhaohui | and Chen Enyong)” gen. prep. in glycerol by Enyong Chen (TAAHU). ***Paratypes***: 5 males, 1 female, Sejila Mountain, Linzhi City, Xizang, China, 29°37'2"N, 94°38'30"E, 4-X-2020, h [Altitude] 4500 m (coll. Pan Zhaohui | and Chen Enyong), unique numbers: STS-32784, STS-32786, STS-32789–32792, gen. preps. in glycerol by Enyong Chen (TAAHU).

##### Diagnosis.

The new species is externally reminiscent of *D.vignai* but is distinguished by the straight costal margin of the forewing, the more elongate forewing apex, the more diffuse forewing pattern in males, and the longer discal spot of the hindwing. Additionally, compared to *D.vignai*, the reniform stigma of *D.sejilaensis* sp. nov. is positioned closer to the forewing costa, and the pale suffusion on the transverse lines and stigmata are grey whereas they are brown in the congener. The male genital capsule of the new species differs clearly from *D.vignai* in the broader valva with a broader and less down curved cucullus, the shorter but considerably thicker, up-curved digitus (it is down curved in *D.vignai*), the broader sacculus and the less prominent, triangular ventral lobe of the valva (whereas it is more rounded in *D.vignai*). Additionally, the uncus, the penicular lobe and the juxta of *D.sejilaensis* sp. nov. are wider than in *D.vignai*. The phallus of the new species is shorter and broader than in *D.vignai* (in proportion to the genital capsule). The vesicae of the two species are similar but the cornuti are more or less equal in size in *D.sejilaensis* sp. nov. whereas the distal cornuti of *D.vignai* are markedly longer and thicker than the proximal ones. In the female genitalia, *D.sejilaensis* sp. nov. differs from *D.vignai* in the longer apophyses anteriores (in proportion to the ovipositor), the narrower, more asymmetrically sclerotised and sideways curved ductus bursae (it is nearly straight in *D.vignai*), and the straight posterior section of the corpus bursae which is sideways curved in *D.vignai*. The detailed comparison with *D.cerritula* sp. nov. is provided below in the diagnosis of the latter species.

##### Description.

**External morphology of adults** (Figs 1–3). Forewing length 11–12 mm in males (11.5 mm in the holotype) and 13 mm in female. Antenna serrulate in male and filiform in female. Head and thorax covered with long hair-like scales, dark brown with admixture of pale grey. Forewing elongate, narrow, with almost parallel costal and anal margins and convex outer margin. Forewing ground colour dark brown, with intense pale grey suffusion along transverse lines and in subterminal area in female; costal margin with diffuse ochreous-brown or grey spots of various sizes. Forewing pattern diffuse, blackish-brown, more distinct in female. Subbasal line short, indistinct. Antemedial line sinuous, double. Orbicular stigma elliptical, filled with ochreous scales in male and pale grey scales in female. Reniform stigma narrow, filled with pale grey scales in female. Postmedial line medially curved, serrulate in female. Subterminal line interrupted into row of indistinct dash-like spots of various sizes between veins. Terminal line interrupted into blackish spots between veins. Outer margin edged with rusty-brown scales. Forewing cilia long, dark brown. Hindwing creamy with greyish-brown suffusion subterminally and terminally and along costal and anal margins. Discal spot large, falcate. Hindwing cilia long, creamy with admixture of brownish-grey scales. Abdomen covered with long hair-like scales, uniform brown. **Male genitalia** (Figs 9, 10). Tegumen short with large trapezoid penicular lobes. Vinculum longer than tegumen, robust, U-shaped. Valva lobular with heavily sclerotised costa and weakly sclerotised, short, broadly triangular and apically rounded ventral lobe. Digitus robust, directed distally, with up curved and distally tapered distal section. Cucullus short, trapezoid, densely covered with hair-like setae. Sacculus short but broad, elliptical. Clasper slightly curved and dilated distally, with very short, tubercle-like harpe. Uncus short but broad, triangular with rounded apex, dorso-ventrally flattened, weakly sclerotised. Juxta broad, shield-like, bearing broad, triangular and apically pointed, heavily sclerotised medial process posteriorly. Anellus weakly granulose. Phallus broad with rounded coecum, somewhat dilated distally. Main chamber of vesica somewhat shorter than phallus, tapered distally, directed ventro-distally, weakly granulose, with short semiglobular dorsal subbasal diverticulum and lengthwise cluster of 6–7 small but robust spike-like cornuti dorso-medially. **Female genitalia** (Fig. 15). Anterior section of corpus bursae membranous, dilated, teardrop-shaped. Posterior section of corpus bursae equal in width to anterior section of ductus bursae, membranous, tubular. Appendix bursae short and narrow, conical, positioned postero-laterally on right side at junction with ductus bursae. Ductus bursae short, its anterior section heavily sclerotised, dorso-ventrally flattened, strongly curved sideways to the right. Posterior section of ductus bursae funnel-like, bearing short, band-shaped antevaginal plate. Ostium bursae broad. Ovipositor short, broad, conical. Apophyses long and thin, apophysis anterioris slightly shorter than apophysis posterioris. Papilla analis setose.

##### Distribution.

The new species is known only from Sejila Mountain in southern Xizang Province of China.

##### Etymology.

The specific epithet refers to the type locality.

#### ﻿ Dasypolia (Tatsipolia) cerritula sp. nov.

Taxon classificationAnimaliaLepidopteraNoctuidae

41B36FA6-01B4-55F3-B71E-E5E5131E106B

https://zoobank.org/CC24CD6D-E613-4517-8E36-9FD1FCE81CE3

Figs 4 , 11

##### Type material.

***Holotype*** (Figs 4, 11): male, “STS-32788 | Sejila Mountain, Linzhi City, | Xizang, China, | N:29°37'2" | E:94°38'30" | 4-X-2020 | h [Altitude] 4500 m (coll. Pan Zhaohui | and Chen Enyong)” gen. prep. in glycerol by Enyong Chen (TAAHU).

##### Diagnosis.

The new species is very similar to the sympatric *D.sejilaensis* sp. nov. but differs in the greyish-brown hindwing with a smaller and rounded discal spot whereas the hindwing of *D.sejilaensis* sp. nov. is creamy with intense greyish suffusion outwardly and along the costal and the anal margins, and the discal spot is large and falcate. The abdomen of *D.cerritula* sp. nov. is covered with black hair-like scales medially and distally whereas it is monotonous brown in *D.sejilaensis* sp. nov. Compared to *D.sejilaensis* sp. nov., the male genital capsule of *D.cerritula* sp. nov. has a narrower uncus, a larger penicular lobe with a more elongated posterior corner, and a shorter valva with a markedly broader cucullus densely covered with more robust setae. Additionally, the digitus of *D.cerritula* sp. nov. is shorter and narrower than in *D.sejilaensis* sp. nov., the ventral lobe of the valva is conspicuously narrower and shorter, the harpe is absent (it is present in *D.sejilaensis* sp. nov.), and the juxta is narrower and bears a somewhat shorter and basally broader posterior medial process. The phalli of the two species display no remarkable differences. The vesica of *D.cerritula* sp. nov. is similar to that of *D.sejilaensis* sp. nov. but differs in the presence of an additional small cluster of small spine-like cornuti ventrally, and the dorsal cluster consisting of markedly larger cornuti.

##### Description.

**External morphology of adult** (Fig. 4). Forewing length 11.5 mm in holotype male. Male antenna serrulate. Head and thorax covered with long hair-like scales, dark brown with admixture of pale grey. Forewing elongate, narrow, with almost parallel costal and anal margins and convex outer margin. Forewing ground colour dark brown with black suffusion in medial area; costal margin with diffuse pale brown spots of various sizes. Forewing pattern diffuse, blackish-brown. Subbasal line short, indistinct. Subbasal lengthwise dash narrow, diffuse. Antemedial line sinuous, with pale brown suffusion inwardly. Orbicular stigma elliptical, filled with pale brown scales. Reniform stigma narrow, filled with pale brown scales. Postmedial line medially curved, slightly dentate posteriorly. Subterminal line interrupted into row of blackish cuneal spots of various sizes between veins. Terminal line indistinct, interrupted into small diffuse blackish spots between veins. Outer margin edged with rusty-brown scales. Forewing cilia long, dark brown. Hindwing brown with somewhat paler subbasal and medial areas. Discal spot small, rounded, diffuse. Outer margin edged with rusty-brown scales. Hindwing cilia long, creamy with admixture of brownish-grey scales. Abdomen covered with long hair-like scales, brown with strong admixture of black scales medially and posteriorly.

**Male genitalia** (Fig. 11). Tegumen short, penicular lobe large, trapezoid with elongate posterior corner. Vinculum longer than tegumen, robust, U-shaped. Valva lobular with heavily sclerotised costa and short, triangular, moderately sclerotised ventral lobe. Digitus heavily sclerotised, directed ventro-distally, with up curved and apically pointed distal section. Cucullus broad, rounded, densely covered with robust spine-like setae. Sacculus short but broad, elliptical. Clasper slightly curved and dilated distally, without harpe. Uncus short but broad, arrowhead-shaped with rounded apex, dorso-ventrally flattened, weakly sclerotised. Juxta broad, rectangular with rounded corners, bearing broad, triangular and apically pointed, heavily sclerotised medial process posteriorly. Anellus weakly granulose. Phallus broad with rounded coecum, somewhat dilated distally. Main chamber of vesica somewhat shorter than phallus, tapered distally, directed ventro-distally, weakly granulose, with short semiglobular dorsal subbasal diverticulum and two lengthwise clusters of cornuti medially: dorsal one consisting of five robust, slightly curved spike-like cornuti, and ventral one consisting of four smaller spine-like cornuti.

**Female** unknown.

##### Distribution.

The new species is known only from Sejila Mountain in southern Xizang Province of China.

##### Etymology.

In Latin, ‘cerritulus’ means ‘weird.’ The specific epithet refers to the unusual cucullus densely covered with robust, spine-like setae.

#### ﻿ Dasypolia (Tatsipolia) vignai

Taxon classificationAnimaliaLepidopteraNoctuidae

L. Ronkay & Zilli, 1992

4E220012-8906-5FAE-822C-7B63B37C07D1

**Figures 12–14.**
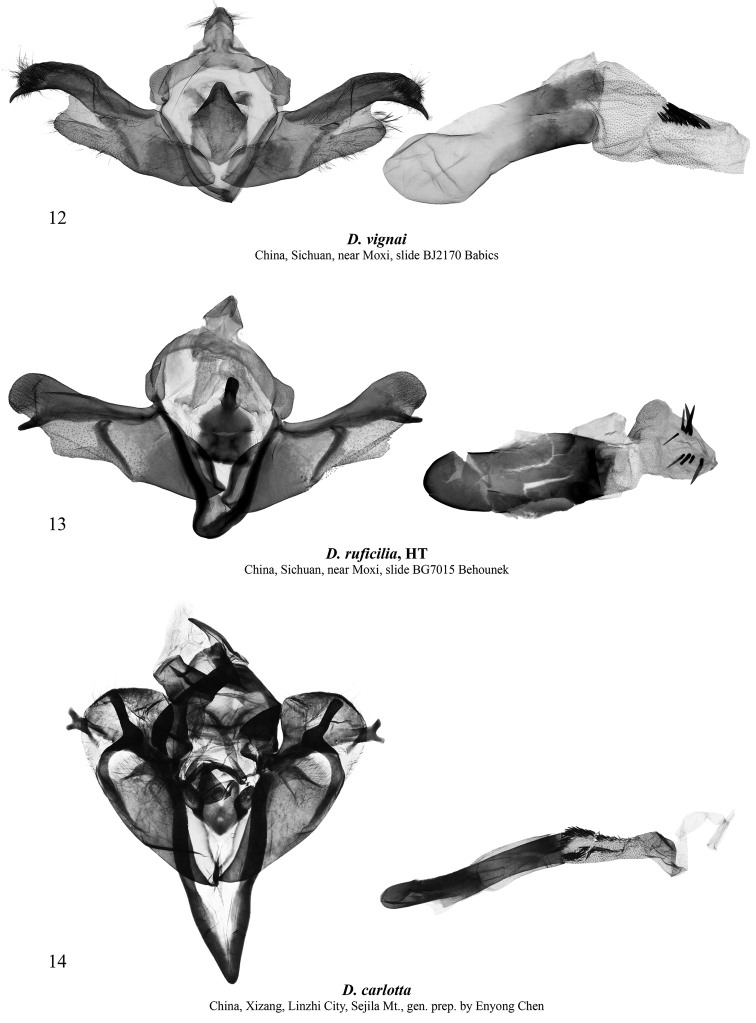
*Dasypolia* (*Tatsipolia* and *Auropolia*) spp., male genitalia. Depositories of the specimens dissected: **12** in AFM**13** in ZSM**14** in TAAHU.

Figs 5 , 6 , 12 , 16

Dasypolia (Sinipolia) vignai Ronkay & Zilli, 1992: 500, fig. 12 (female genitalia), pl. O: fig. 11 (adult) (Type locality: “China – Sichuan, Gongga Shan 4100 m ... Yantsoko Valley”).

##### Type material examined.

***Holotype*** (Figs 6, 16): female, “China – Sichuan | Gongga Shan 4100 m | vers. N 27.V.[19]90 | Yantso Ko Valley, A. Vigna Taglianti leg.” | “Holotypus | *Dasypolia* | *vignai* sp. nov. | Ronkay & Zilli | det. L. Ronkay/91” / “3865♀ | gen. prep. No. | det.L.Ronkay” (HNHM).

##### Additional material examined.

1 male, China, W Sichuan, near Moxi, H-3954 m, 07.X.2011, 29°53.097'N, 102°00.459'E, A. Floriani leg., gen. prep. No.: JB2170 (prepared by Babics) (AFM).

##### Diagnosis.

The forewing length is 12 mm in both sexes. The species is externally similar to *D.sejilaensis* sp. nov. and *D.cerritula* sp. nov. but is distinguished by the slightly convex costal margin of the forewing, the shorter and more rounded forewing apex, the more distinct forewing pattern in males, and the shorter discal spot of the hindwing. Additionally, compared to the congeners, the reniform stigma *D.vignai* is positioned more inwardly from the forewing costa. The male genitalia of *D.vignai* differ clearly from other species of the subgenus Tatsipolia in the narrow cucullus, the long, down curved and apically pointed digitus, and the large and rounded ventral lobe of the valva. The detailed comparison with *D.sejilaensis* sp. nov. is provided above in the diagnosis of the latter species.

##### Distribution.

The species is known from two localities in Sichuan Province, south-western China.

**Figures 15–16.**
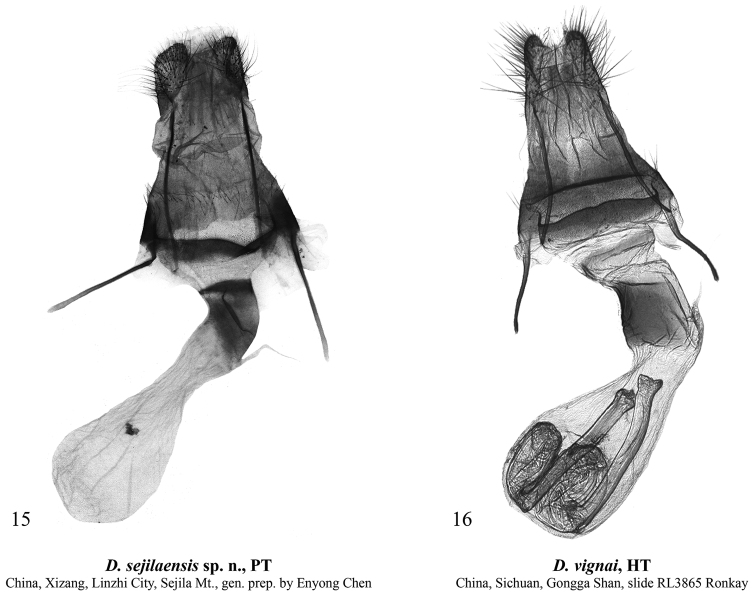
Dasypolia (Tatsipolia) spp., female genitalia. Depositories of the specimens dissected: **15** in TAAHU**16** in HNHM (photo by B. Tóth).

#### ﻿ Dasypolia (Tatsipolia) ruficilia

Taxon classificationAnimaliaLepidopteraNoctuidae

Benedek, Behounek, Floriani & Saldaitis, 2011

2401370D-08AC-5544-B73B-D42F5145C44D

Figs 7 , 13

Dasypolia (Tatsipolia) ruficilia Benedek, Behounek, Floriani & Saldaitis, 2011: 108, fig. 2 (male genitalia), pl. 15: fig. 4 (adult) (Type locality: “China, W. Sichuan, Kangding, near Zheduo Pass, 3700–4200 m”).

##### Type material examined.

***Holotype*** (Figs 7, 13): male, “China, W.Sichuan | Kangding, near | Zheduo Pass | h-3700–4200 m | 14.10.2009 | Floriani & Saldaitis leg.” / red label “Holotypus | *Dasypoliaruficilia* | Benedek, Behounek, Floriani & | Saldaitis sp. nov., 2011 | *Esperiana*, Buchreihe zur | Entomologie, 16: 108,” gen. prep. No.: BG7015 (prepared by Behounek) (ZSM).

##### Diagnosis.

The forewing length is 12 mm in the holotype male. The species externally differs from other members of the subgenus Tatsipolia in the monotonous pale brown forewing colouration and reddish-brown forewing cilia. The male genital capsule is characterised by the broad, trapezoid and weakly setose cucullus and the nearly straight digitus. The phallus of *D.ruficilia* is distally tapered whereas it is distally somewhat dilated in other species in the subgenus. The vesica of *D.ruficilia* bears two short lengthwise clusters consisting of four long and thin spine-like cornuti each, whereas the clusters of other Dasypolia (Tatsipolia) species consist of more robust cornuti.

The female is unknown.

##### Distribution.

The species is known only from its type locality in the western Sichuan Province of China (Benedek et al. 2011).

### SubgenusAuropolia Hreblay & L. Ronkay, 1999

#### ﻿ Dasypolia (Auropolia) carlotta

Taxon classificationAnimaliaLepidopteraNoctuidae

Floriani, Benedek, Behounek & Saldaitis, 2011

B1A3A321-B1CF-58E1-8066-E34DB1B325E0

Figs 8 , 14

Dasypolia (Auropolia) carlotta Floriani, Benedek, Behounek & Saldaitis in Benedek et al. 2011: 111, fig. 6 (male and female genitalia), pl. 14: figs 7–9 (adults) (Type locality: “China, W. Sichuan, Kangding, near Zheduo Pass, 3700–4200 m”).

##### Material examined.

1 male, Sejila Mountain, Linzhi City, Xizang, China, 29°39'4.5"N, 94°42'51.5"E, 15-XI-2021, [Altitude] h 4160 (coll. Enyong Chen, Xiongyan Yin), unique number: STS-40035, gen. prep. in glycerol by Enyong Chen (TAAHU).

##### Distribution.

The species is known from south-western China: western Sichuan (Benedek et al. 2011), north-western Yunnan (Benedek and Saldaitis 2014) and southern Xizang (new record).

## Supplementary Material

XML Treatment for Subgenus Tatsipolia

XML Treatment for Dasypolia (Tatsipolia) sejilaensis

XML Treatment for Dasypolia (Tatsipolia) cerritula

XML Treatment for Dasypolia (Tatsipolia) vignai

XML Treatment for Dasypolia (Tatsipolia) ruficilia

XML Treatment for Dasypolia (Auropolia) carlotta
